# Effects of Pyrolysis Temperature on Pore Structure, Carbon Disorder, and Thermal Transport in Rice Husk Char/PVDF Films

**DOI:** 10.3390/nano16211374

**Published:** 2026-10-27

**Authors:** Kaiyan Cao, Kun Cao, Yiran Wang, Fanghua Tian, Chao Zhou, Qizhong Zhao, Minxia Fang, Yu Wang, Yin Zhang, Sen Yang

**Affiliations:** MOE Key Laboratory for Nonequilibrium Synthesis and Modulation of Condensed Matter, School of Physics, Xi’an Jiaotong University, Xi’an 710049, China; caokaiyan@xjtu.edu.cn (K.C.); 18686052674@163.com (K.C.); wyr050415@stu.xjtu.edu.cn (Y.W.); tfh2017@xjtu.edu.cn (F.T.); chao.zhou@xjtu.edu.cn (C.Z.); zhaoqizhong@xjtu.edu.cn (Q.Z.); minxia.fang@xjtu.edu.cn (M.F.); yuwang@mail.xjtu.edu.cn (Y.W.); yangsen@mail.xjtu.edu.cn (S.Y.)

**Keywords:** biomass-derived carbon, rice husk char, pyrolysis temperature, thermal conductivity, porous composite film

## Abstract

Low-cost, sustainable thermal insulation materials are needed for energy-efficient thermal management. Here, porous rice husk char (RHC) was produced from agricultural waste by pyrolysis at 300, 500, and 700 °C. RHC/polyvinylidene fluoride (PVDF) composite films were then prepared by blade coating where feasible. We examined how pyrolysis temperature affected char structure, carbon disorder, film morphology, and thermal transport. RHC-300-16 did not form an intact self-supporting film under the processing conditions used. RHC-700-16 contained coarser agglomerates and larger visible voids than RHC-500-16. RHC-500-16 exhibited a higher BET specific surface area (3.1347 m^2^·g^−1^) and total pore volume (0.007716 cm^3^·g^−1^) than RHC-700-16 (1.9471 m2·g^−1^ and 0.003153 cm^3^·g^−1^, respectively), with average BJH pore diameters of 33.30 and 21.66 nm, respectively. RHC-500-16 had a room-temperature thermal conductivity of 0.059 W·m^−1^·K^−1^. In a qualitative 100 °C heating-stage test, RHC-500-16 exhibited a lower surface temperature than a thickness-matched paper control. The measured thermal conductivity was associated with the combined effects of pore characteristics, film morphology, interparticle contacts, and carbon structure. These results link pyrolysis temperature to char structure and composite-film thermal conductivity in biomass-derived insulation films.

## 1. Introduction

Rising global energy demand and growing requirements for thermal management have intensified interest in effective insulation for buildings, electronic protection, and wearable systems. The buildings and construction sector accounts for approximately 37% of global CO_2_ emissions [1], making reductions in heat loss and operational energy demand important. Sustainable insulation materials that combine low thermal conductivity with practical processing are therefore important for both energy efficiency and emissions reduction.

Mineral wool, polystyrene and phenolic foams, and foam glass are widely used, but their environmental burden, processing energy, service-temperature range, or end-of-life options can limit particular applications [2,3]. Silica aerogels and vacuum insulation panels can provide much lower thermal conductivity, although cost, fragility, edge effects, moisture sensitivity, and manufacturing complexity remain barriers to broad deployment [2,3]. These trade-offs continue to motivate low-cost, scalable, and mechanically compliant alternatives.

Beyond conventional insulation, alternative materials derived from waste and renewable resources have attracted increasing attention. Fly ash, particularly hollow cenospheres, offers low density and favorable thermal resistance, while recycled concrete powder has been incorporated into foamed concrete for waste valorization and thermal insulation [4,5,6]. Natural fibers such as hemp and flax provide renewable insulation options, whereas recycled microplastics have also been explored in thermal-insulating composites [7,8]. These approaches highlight the potential of converting diverse waste streams into functional insulation materials within a circular-economy framework.

Biomass-derived chars are promising insulation components because they can combine low density, hierarchical porosity, and renewable feedstocks. Pyrolysis converts lignocellulosic biomass into carbon-rich frameworks whose pore structure and solid-phase order depend strongly on processing conditions [9]. Rice husk (RH) is an abundant agricultural byproduct that contains both lignocellulosic matter and biogenic silica, enabling routes to porous carbon and silica-based materials [10,11,12].

Recent research on rice-husk insulation spans several material platforms [13,14,15,16,17,18,19,20]. Rice-husk-earth panels, rice husk ash (RHA)-containing mortars, and rice-husk-derived silica-aerogel/cement composites have provided routes to reduce density or heat transfer in building envelopes [21,22,23]. More recent studies have developed rice-husk/reed-fiber boards, recycled-cellulose/rice-husk panels, and RHA-based multifunctional foamed concrete [24,25,26]. Work published in 2025-2026 has also applied machine-learning-guided processing to hydrophobic RHA-derived silica aerogels and incorporated rice-husk-derived biosilica into polyurethane foams or recycled-glass foams [27,28,29,30]. Current research priorities include pore-scale control, moisture resistance, interfacial compatibility, fire performance, durability, and low-energy scalable processing.

Pyrolysis temperature strongly affects char yield, pore development, surface chemistry, aromatic ordering, and other biochar properties [9,31]. Studies of rice-husk biochar/polymer composites have further shown that pyrolysis conditions and filler dispersion influence composite thermal, mechanical, and fire-related behavior [32,33]. However, direct links among pyrolysis temperature, char pore structure, carbon disorder, and heat transport remain underexplored in thin composite films made by scalable coating processes.

In this study, porous RHC was prepared by controlled pyrolysis at 300, 500, and 700 °C. RHC/PVDF composite-film formation was then attempted by blade coating. The structural evolution of RHC was compared across all three pyrolysis temperatures, whereas film morphology and thermal performance were evaluated only for the successfully formed RHC-500-16 and RHC-700-16 films.

## 2. Materials and Methods

### 2.1. Preparation and Pyrolysis of Rice Husk Char

Rice husk samples obtained from rural area of Chongqing, China were ultrasonically cleaned with deionized water and ethanol four to six times to remove impurities and were then dried at 55 °C. The dried RH was pulverized and passed through 60–100 mesh sieves to obtain particles of 147–250 μm. Separate batches of RH powder were then carbonized in a tube furnace under argon at 300, 500, or 700 °C for 1 h, using a heating rate of 5 °C·min^−1^. After cooling naturally to room temperature, the resulting chars were designated RHC-300, RHC-500, and RHC-700, respectively.

### 2.2. Fabrication of RHC/PVDF Films

For each composition, 200 mg of RHC powder was mixed with 12.5 mg of PVDF (HSV900, Zhengzhou Jinghong New Energy Technology Co., Ltd., Zhengzhou, China) and ground in a mortar for 1 h, corresponding to an RHC:PVDF mass ratio of 16:1. This ratio was selected from preliminary formulation screening of 4:1, 8:1, and 16:1 compositions, in which the 16:1 formulation provided a favorable balance between film formation and low thermal conductivity under the tested processing conditions. It should be noted that this composition was selected as an experimentally suitable formulation rather than as a fully optimized RHC/PVDF ratio. N-Methyl-2-pyrrolidone (NMP; Sinopharm Chemical Reagent Co., Ltd., Shanghai, China) was then added dropwise while grinding continued until a homogeneous slurry formed. The slurry was cast onto a glass substrate, leveled by blade coating, and dried at 80 °C. The dried products were released from the glass with deionized water and designated RHC-300-16, RHC-500-16, and RHC-700-16. Under these conditions, RHC-300-16 did not form an intact self-supporting film.

### 2.3. Characterization

Material morphology, phase structure, chemical bonding, carbon structure, and pore characteristics were examined by scanning electron microscopy (SEM; VE-9800S, KEYENCE Corporation, Osaka, Japan), X-ray diffraction (XRD; D8 ADVANCE, Bruker AXS GmbH, Karlsruhe, Germany), Raman spectroscopy (DXR2xi, Thermo Fisher Scientific, Madison, WI, USA), Fourier-transform infrared spectroscopy (FTIR; VERTEX 70, Bruker Optik GmbH, Ettlingen, Germany), and N_2_ adsorption–desorption analysis (ASAP 2020 Plus HD88, Micromeritics Instrument Corporation, Norcross, GA, USA). The XRD measurements were performed using Cu Kα radiation (λ = 1.5406 Å) generated by a 2.2 kW ceramic X-ray tube with a focal spot of 0.4 × 12 mm. SEM observations were conducted at an accelerating voltage of 0.5–0.7 kV under a chamber vacuum of approximately 9 × 10^−3^ Pa. The magnification was adjusted according to the feature size and observation area, as indicated by the scale bars in the corresponding micrographs.

The thermal behavior of RH and RHC was characterized using a simultaneous thermal analyzer (TGA/DSC3+, Mettler-Toledo AG, Schwerzenbach, Switzerland). For each measurement, the sample was placed in a crucible, and comparable sample masses were used to minimize variations associated with sample loading. The samples were heated from room temperature to 800 °C at a rate of 10 °C·min^−1^. TG/DTG measurements of RH were conducted under N_2_ to characterize its thermal decomposition under an inert atmosphere and to provide a basis for selecting the pyrolysis temperatures. In contrast, the RHC samples, which had already undergone pyrolysis, were measured in air to evaluate their oxidative thermal stability and compare their characteristic oxidation behavior after carbonization at different temperatures. The thermal conductivity of the composite films was measured at room temperature using a laser flash analyzer (LFA 447, Netzsch-Gerätebau GmbH, Selb, Germany). Before measurement, the samples were prepared as circular disks with a diameter of 12.7 mm. The thermal diffusivity α was directly determined by the laser flash method. The thermal conductivity K was subsequently calculated using *K* = *αC_P_ρ*, Where *ρ* and *C_P_* are the density and specific heat capacity of the specimen, respectively. A polyimide (VESPEL, DuPont, Wilmington, DE, USA) disk with a diameter of 12.7 mm was used as the reference material for comparative measurement. Data are presented based on three measurements performed at each specimen.

To further evaluate the transient heat-transfer behavior of the composite film, an RHC-500-16 slurry was coated onto a quartz glass plate over an area of 60 mm × 60 mm, with a dried film thickness of approximately 0.5 mm. The coated samples were placed on a heating stage maintained at either 100 or 350 °C. The temperature at the upper surface of the film was monitored using a Type-K thermocouple (TA611B, TASI, Suzhou, China) for 300 s, with data recorded at 5 s intervals.

The response of RHC-500-16 to direct flame exposure was assessed qualitatively using an alcohol lamp. This experiment was intended only to observe the short-term response of the film to direct flame and was not conducted according to a standardized fire-resistance or flame-retardancy testing protocol. The sample was exposed for 10 s to the outer flame (approximately 400–500 °C) at a flame height of approximately 4 cm. After the flame was removed, the persistence of visible glowing and the gross sample morphology were recorded.

## 3. Results and Discussion

### 3.1. Pyrolysis Behavior and Thermal Stability

**Figure 1:**
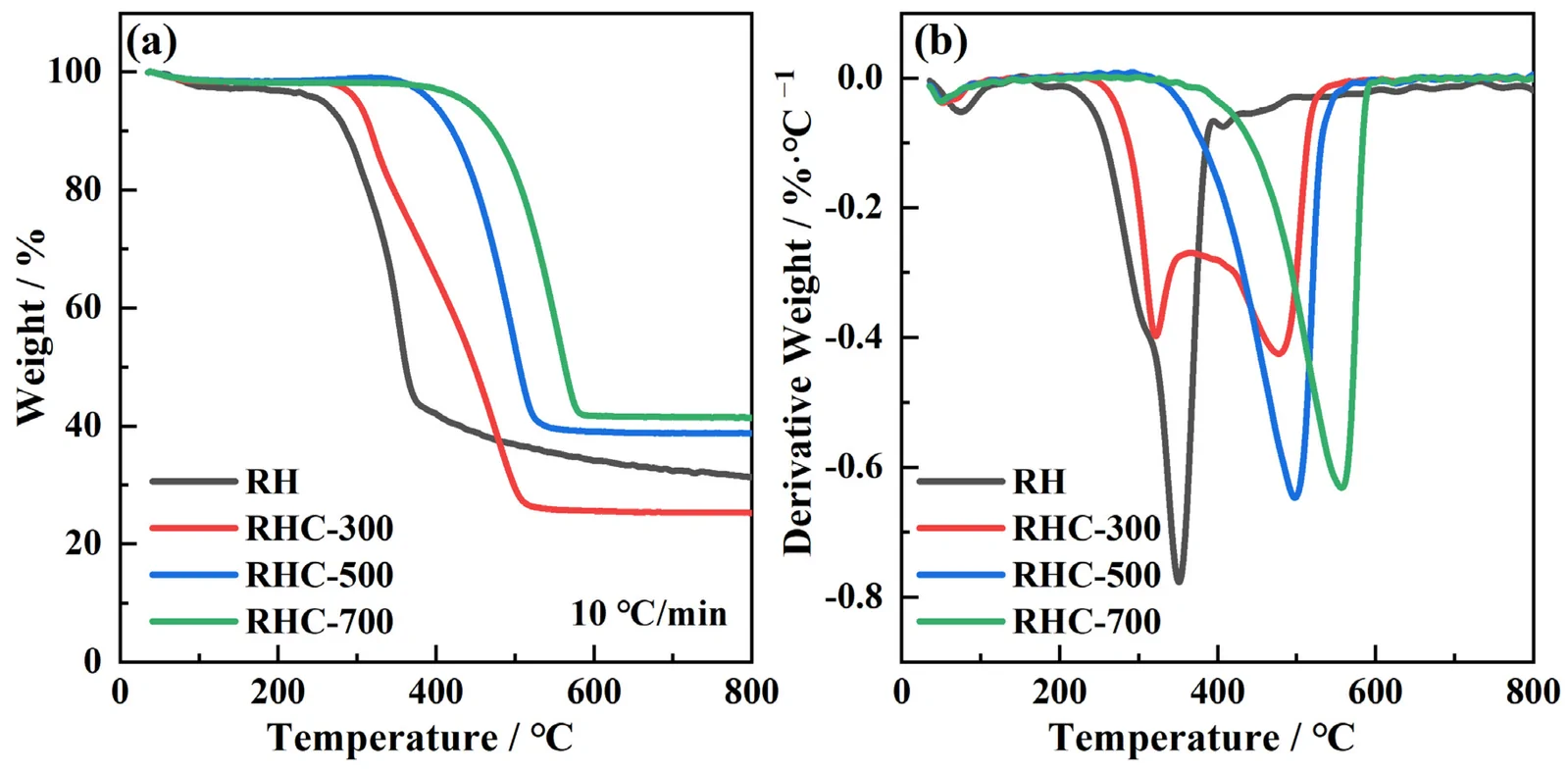
TG and DTG curves of raw RH and rice husk chars prepared at 300, 500, and 700 °C: (**a**) TG curves and (**b**) DTG curves. RH was measured under N_2_ to characterize inert-atmosphere thermal decomposition, whereas the RHC samples were measured in air to evaluate oxidative thermal stability. The heating rate was 10 °C·min^−1^.

Figure 1 compares the thermogravimetric (TG) and derivative thermogravimetric (DTG) profiles of RH and the three chars. Under nitrogen, RH showed minor mass loss below 220 °C, followed by rapid devolatilization between approximately 220 and 400 °C. The DTG profile shows a shoulder near 307 °C and a main feature near 351 °C within the major devolatilization region. These features are consistent with overlapping thermal degradation of the lignocellulosic components, particularly hemicellulose- and cellulose-rich fractions; however, they cannot be uniquely assigned to individual components because no evolved-gas analysis was performed. Above 400 °C, the mass-loss rate decreased as carbonization proceeded. These features guided the selection of 300, 500, and 700 °C as the holding temperatures.

Because the RHC samples had already been carbonized, their TG/DTG measurements in air were used to assess oxidative thermal stability rather than pyrolysis behavior. Accordingly, their mass-loss features represent oxidation of the carbonized material and should be distinguished from the inert-atmosphere decomposition of raw RH. RHC-300 showed two major DTG features near 322 and 477 °C, whereas the main high-temperature features of RHC-500 and RHC-700 occurred near 498 and 557 °C, respectively. After pyrolysis at 500 and 700 °C, the main oxidation peaks shifted to higher temperatures, suggesting improved oxidative stability relative to RHC-300. All curves reached plateaus above 600 °C.

Table 1 shows an overall decrease in char yield with increasing pyrolysis temperature. A pronounced decrease was observed between 300 and 500 °C, whereas the yields obtained at 500 and 700 °C were relatively similar. Because these values were obtained from individual pyrolysis batches rather than replicate experiments, they are used here to indicate the overall trend rather than for statistical comparison.

### 3.2. Evolution of Chemical and Carbon Structures

**Figure 2:**
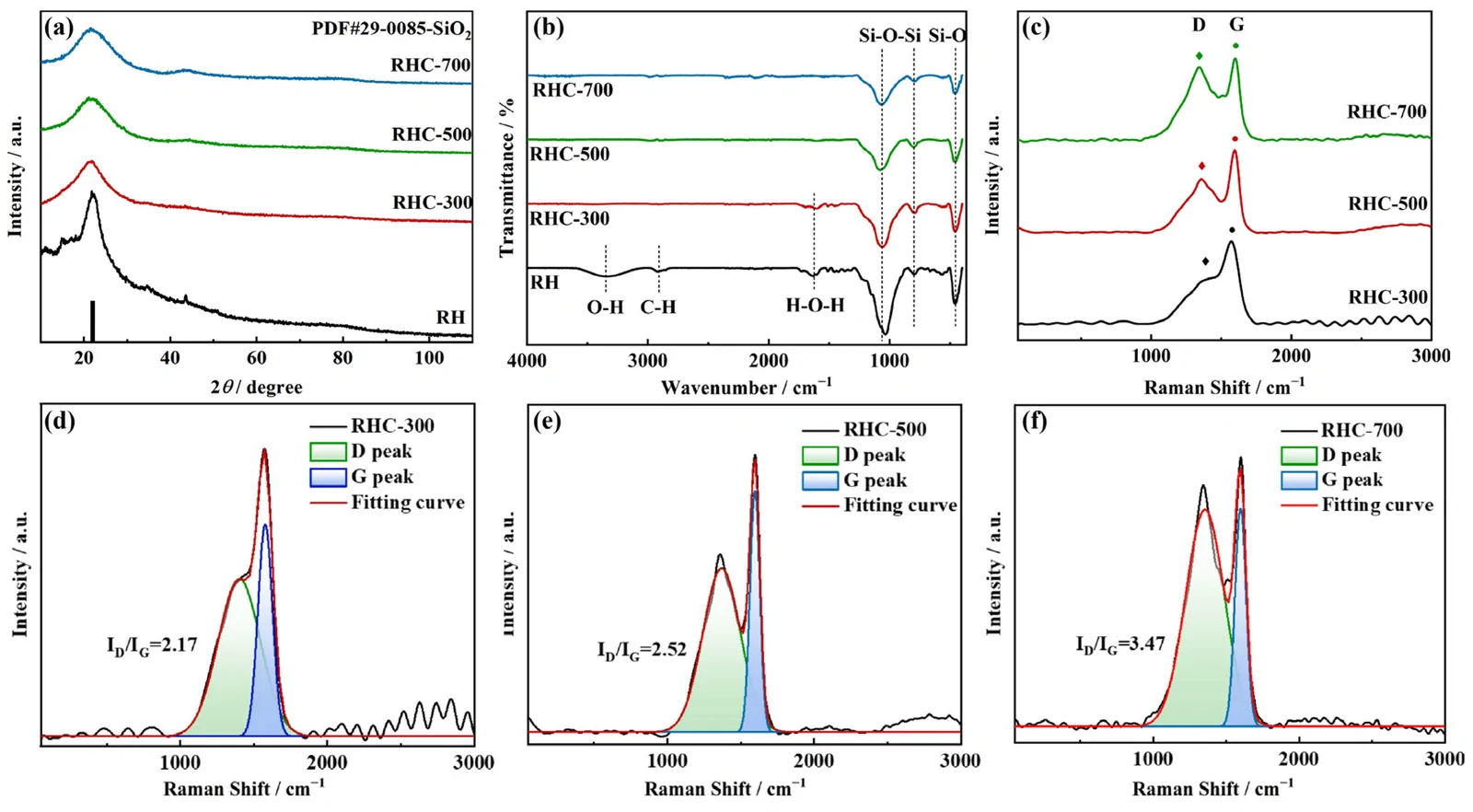
Characterization of RH and RHC by XRD, FTIR, and Raman: (**a**) XRD patterns; (**b**) FTIR spectra; (**c**) Raman spectra; and Gaussian fitting of the D and G bands for (**d**–**f**) RHC-300, RHC-500, and RHC-700, respectively.

Figure 2a shows the XRD patterns of raw RH and the RHC samples carbonized at different temperatures. None of the four patterns contains a sharp crystalline reflection over 2θ = 10–110°. Instead, a broad feature near 22° may arise from amorphous silica or disordered carbon. The absence of sharp reflections indicates that the detected phases are predominantly poorly crystalline or amorphous. Changes in the broad feature with temperature may reflect variations in composition and short-range order within the silica-containing carbon matrix. A weak feature near 43° in RHC-500 and RHC-700 may be assigned to the (100) reflection of turbostratic carbon. The absence of sharp graphitic reflections indicates limited long-range carbon ordering.

Figure 2b presents the FTIR spectra of raw RH and the RHC samples. Raw RH shows bands at 3347.47 and 2916.55 cm^−1^ that are absent after carbonization. The broad band at 3347.47 cm^−1^ is assigned to O-H stretching in hydroxyl groups and adsorbed water; its disappearance after carbonization at 300 °C suggests a reduction in adsorbed water and hydroxyl-containing groups. The band at 2916.55 cm^−1^ corresponds to aliphatic C-H stretching, and its disappearance after carbonization indicates that the aliphatic functional group structures in the rice husk char decompose as the lignocellulose undergoes demethoxylation and dehydration. These changes correspond to mass loss in the low-temperature region of the thermogravimetric profile. The band near 1630.37 cm^−1^ is mainly assigned to the H-O-H bending vibration of adsorbed water. Its progressive weakening after carbonization is therefore primarily associated with dehydration and reduced water adsorption. Bands near 1072.23 and 799.73 cm^−1^ correspond to asymmetric and symmetric Si-O-Si stretching, respectively, whereas the band at 461.56 cm^−1^ is assigned to Si-O bending. These bands weakened as the temperature increased. The XRD and FTIR results suggest that silica in RH is predominantly amorphous and associated with siloxane groups. The characteristic FTIR bands and their corresponding vibrational assignments are summarized in Table 2.

Figure 2c shows the Raman spectra of the RHC samples. The D-band located at 1350 cm^−1^ arises from the in-plane vibration of disordered graphitic carbon rings, whereas the G-band at 1580 cm^−1^ corresponds to the sp^2^ vibration of crystalline graphitic carbon [34]. Here, the I_D_/I_G_ is calculated from the peak area ratio of the D and G bands, which is used to evaluate the degree of graphitization of the material. As the pyrolysis temperature increased, the D and G bands became more distinct, indicating progressive formation of aromatic carbon structures. The Gaussian fits in Figure 2d–f showed an increase in I_D_/I_G_ with pyrolysis temperature, indicating progressively greater structural disorder in the carbon phase. However, Raman spectroscopy alone does not establish the contribution of this disorder to thermal transport.

Elemental analysis was used to examine changes in the C, H, O, N, and S contents of RH and the RHC samples (Table 3). Carbon content increased from 40.18% in RH to 54.50% in RHC-700 as the carbonization temperature increased. By contrast, H content decreased from 5.32% to 1.00%, and O content decreased from 42.28% to 14.47%. The N and S contents increased slightly after carbonization but remained low. The increase in C content indicates a progressive degree of rice husk carbonization, whereas the decrease in O and H results from the cleavage of crystalline water and chemical bonds with lower bond energies within the rice husks as the temperature rises.

The progressive decrease in O/C indicates deoxygenation of RHC as pyrolysis temperature increased under argon. This decrease also indicates lower hydrophilicity of the RHC samples. The lower H/C ratio at higher pyrolysis temperatures suggests increasing aromatic condensation of the carbon phase. Only RHC-700 had H/C < 0.3, indicating a highly carbonized aromatic structure. The (N + O)/C ratio provides an index of char polarity; its decrease indicates fewer polar functional groups. Thus, increasing pyrolysis temperature reduced the hydrophilicity and polarity of RHC while increasing its aromatic character.

### 3.3. Microstructural Evolution of Rice Husk Char

**Figure 3:**
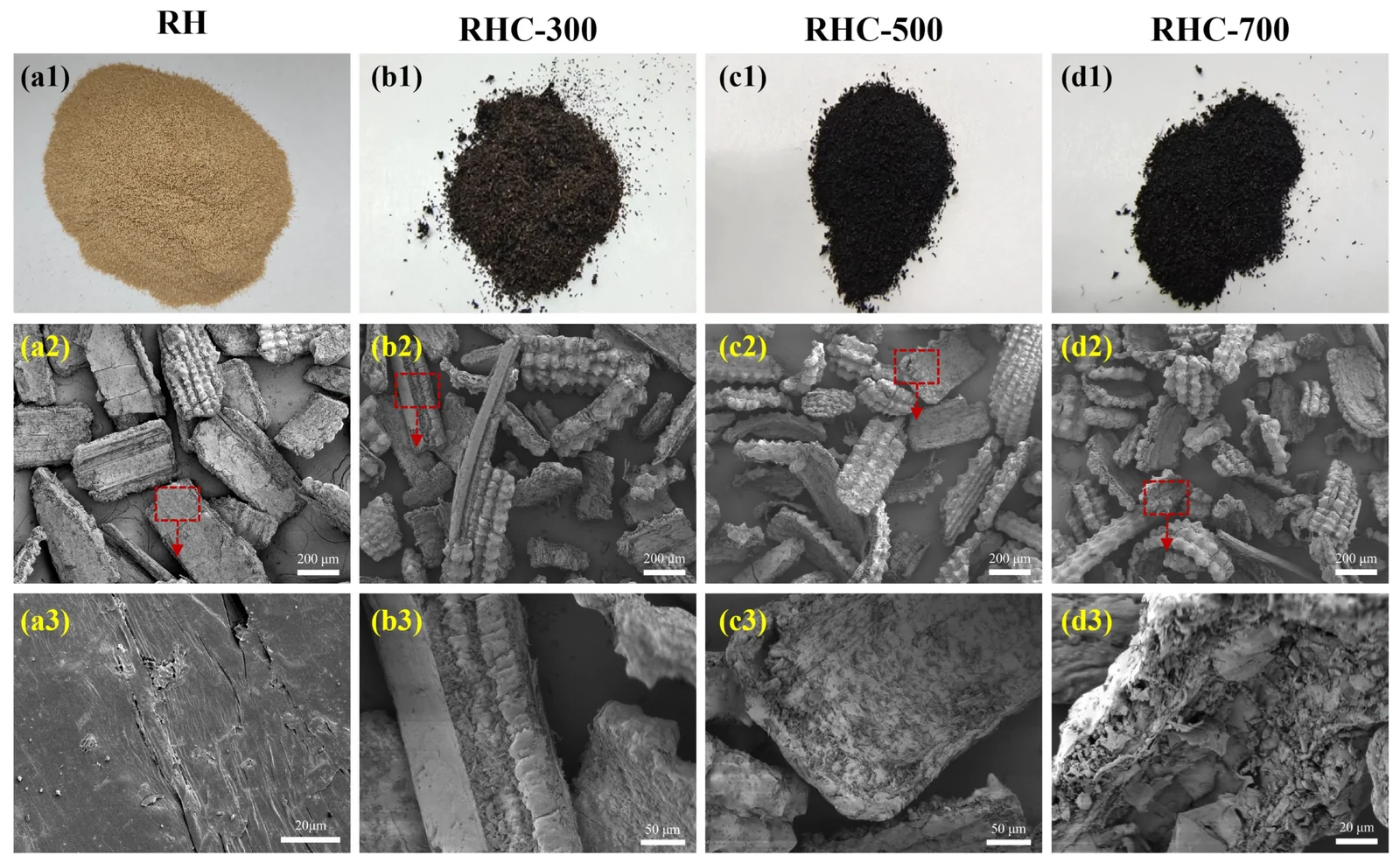
Appearance and morphology of RH and rice husk chars: (**a1**–**d1**) photographs of RH, RHC-300, RHC-500, and RHC-700, respectively; (**a2**–**d2**) corresponding low-magnification SEM images; and (**a3**–**d3**) higher-magnification SEM images of the red-boxed regions in (**a2**–**d2**), respectively.

Figure 3 presents photographs and SEM images of RH and the RHC samples. The raw RH powder consists of yellow particles, which gradually turn black after pyrolytic carbonization. The RHC-300 powder contains some admixed yellow particles, indicating that RH was not completely carbonized at this temperature. SEM images reveal that the RH powder retains a relatively smooth surface; cracks appear on some surfaces of the RHC-300 particles, but their structures remain relatively intact. With increasing carbonization temperature, some RHC-500 and RHC-700 particles become curled, and their surface roughness increases. In combination with the aforementioned char yield and thermogravimetric analysis, increasing temperature intensifies the decomposition of organic matter within the rice husk, further increasing the degree of cellular disruption. Furthermore, as observed in Figure 3(c1–d3), irregular pores appear on the carbonized RH fibers, which are formed by the thermal decomposition of organic matter.

### 3.4. Film Formation, Morphology, and Pore Characteristics

**Figure 4:**
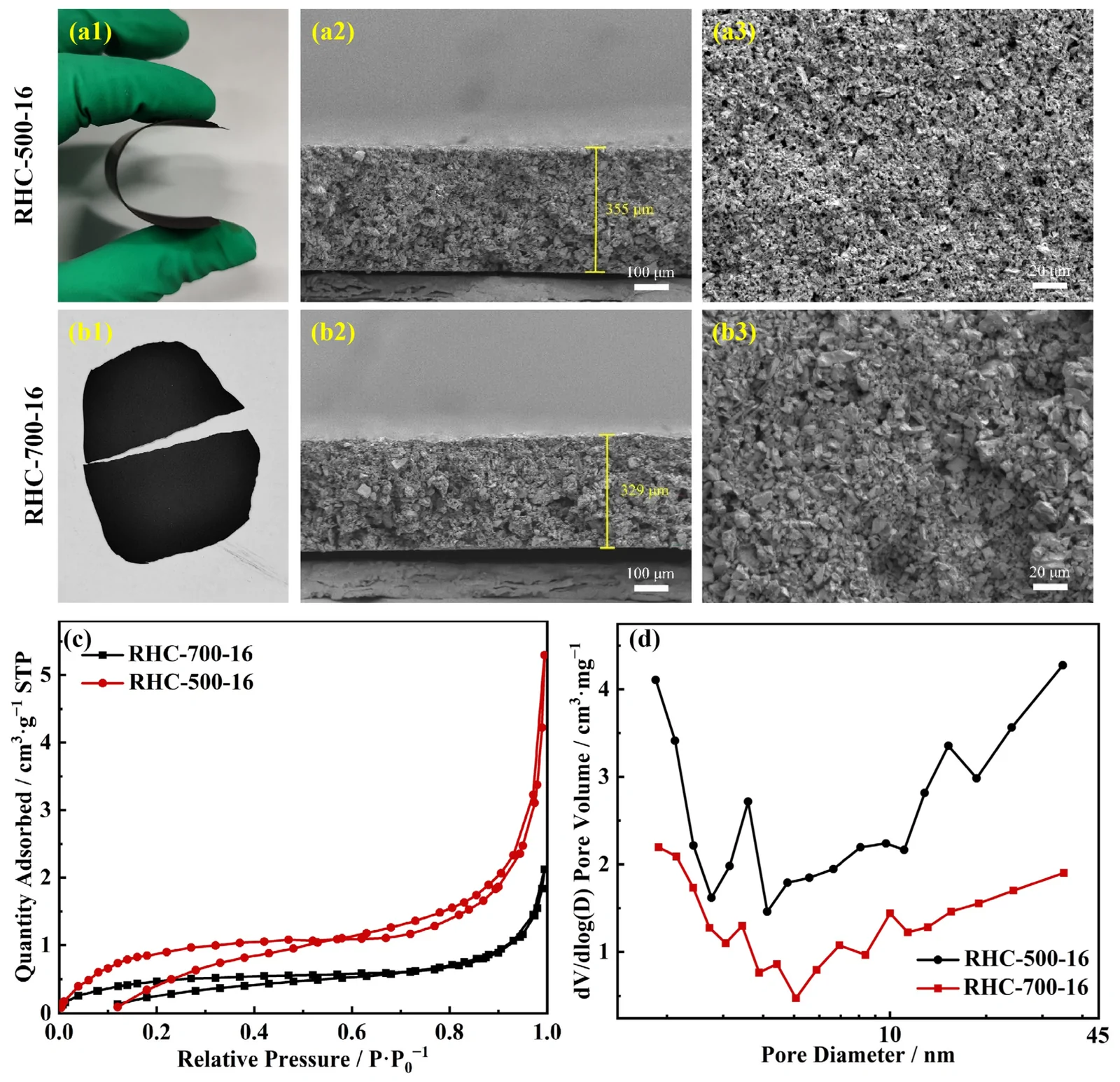
Film appearance, morphology, and pore characteristics: bending photograph, cross-sectional SEM image, and surface SEM image of (**a1**–**a3**) RHC-500-16 and (**b1**–**b3**) RHC-700-16; (**c**) N_2_ adsorption–desorption isotherms; and (**d**) BJH desorption pore-size distributions, dV/dlog(D).

RHC-300-16 did not produce an intact self-supporting film under the processing conditions used and was therefore excluded from subsequent film-level morphology and thermal-performance comparisons. In the qualitative bending demonstration, RHC-500-16 reached the curvature shown in Figure 4(a1) without visible fracture, whereas RHC-700-16 fractured during the initial bend (Figure 4(b1)). This observation provides only a qualitative indication of the different bending responses of the two films and should not be interpreted as a quantitative evaluation of mechanical flexibility. Cross-sectional SEM images show thicknesses of approximately 355 μm for RHC-500-16 and 329 μm for RHC-700-16, a difference of approximately 8% (Figure 4(a2,b2)). Surface images show a finer, more homogeneous texture for RHC-500-16, whereas RHC-700-16 contains coarser agglomerates and larger visible voids (Figure 4(a3,b3)).

The N_2_ adsorption–desorption isotherms and BJH pore-size distributions are shown in Figure 4c,d. RHC-500-16 exhibited a higher BET specific surface area (3.1347 m^2^·g^−1^) and total pore volume (0.007716 cm^3^·g^−1^) than RHC-700-16 (1.9471 m^2^·g^−1^ and 0.003153 cm^3^·g^−1^, respectively). The average BJH pore diameters were 33.30 nm for RHC-500-16 and 21.66 nm for RHC-700-16. These results demonstrate that increasing the pyrolysis temperature from 500 to 700 °C altered the accessible pore structure of the composite films. The SEM images additionally show coarser agglomerates and larger visible voids in RHC-700-16. These features occur at a different length scale from the mesopores characterized by N_2_ adsorption and therefore should not be directly equated with the BJH-derived pore-size parameters. High pyrolysis temperatures can enlarge pores or partially collapse carbon frameworks, depending on the feedstock and processing conditions [31]. In the present samples, the observed coarsening from RHC-500-16 to RHC-700-16 is consistent with this structural evolution.

**Figure 5:**
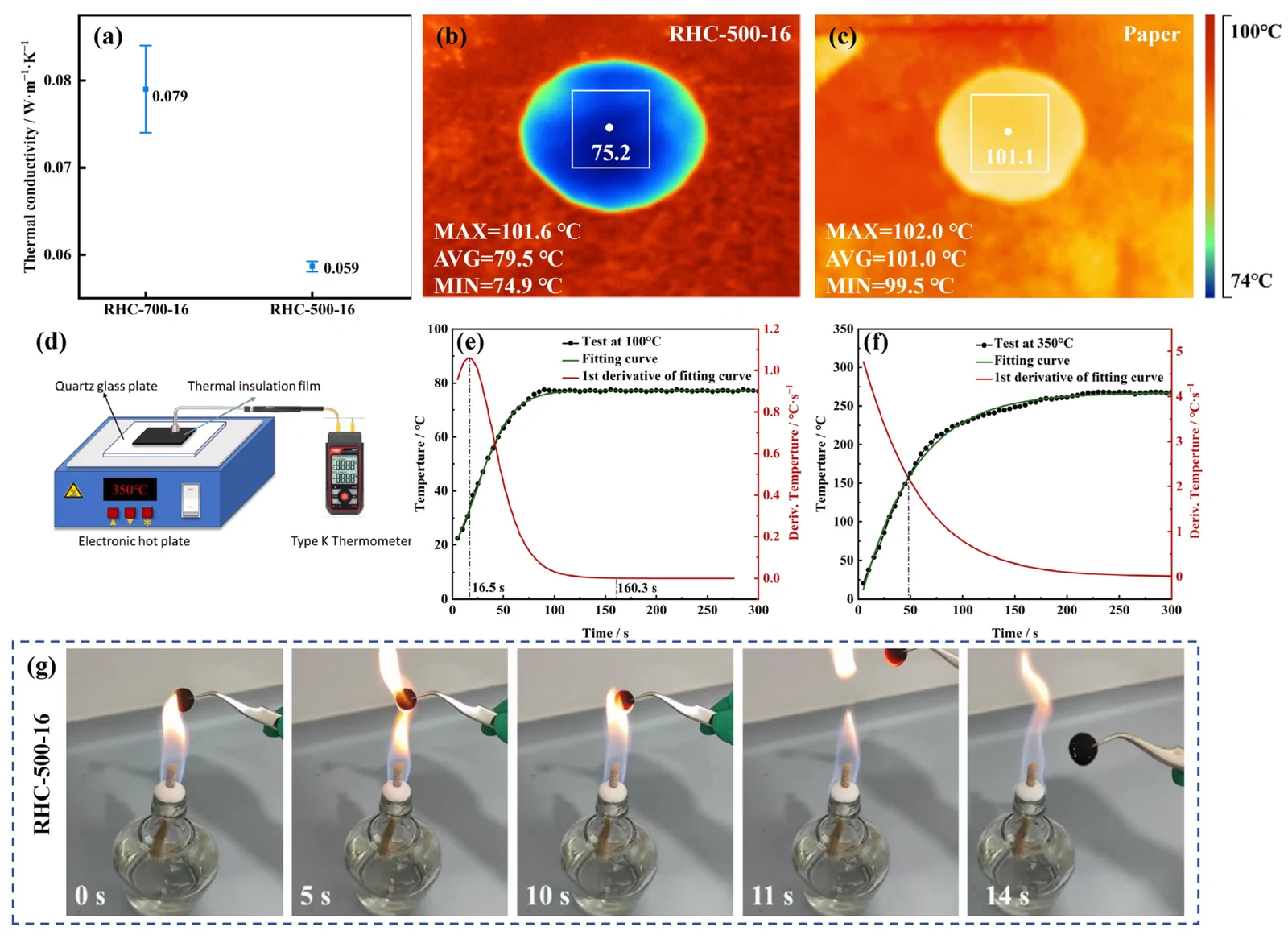
Thermal characterization and qualitative direct-flame exposure of the composite film: (**a**) room-temperature thermal conductivity of RHC-700-16 and RHC-500-16; infrared images of (**b**) RHC-500-16 and (**c**) the paper control on a 100 °C heating stage; (**d**) schematic of the transient heat-transfer experiment; surface-temperature evolution and corresponding fitted curves and first derivatives for RHC-500-16 on heating stages maintained at (**e**) 100 °C and (**f**) 350 °C; and (**g**) qualitative direct-flame exposure of RHC-500-16 from 0 to 14 s. The thermal conductivity values are averages of three measurements performed at different regions of each specimen, and the error bars indicate the variation among these measurements.

As shown in Table 4 and Figure 5a, RHC-500-16 had a thermal conductivity of 0.059 W·m^−1^·K^−1^, compared with 0.079 W·m^−1^·K^−1^ for RHC-700-16. The lower thermal conductivity of RHC-500-16 was associated with its distinct accessible pore characteristics and film morphology. The higher accessible pore volume and distinct pore-size distribution of RHC-500-16 may increase the tortuosity of heat-transfer pathways and contribute to suppression of gaseous heat conduction within the porous film.

### 3.5. Thermal Performance and Qualitative Flame Exposure

For the qualitative infrared heating-stage experiment, RHC-500-16 and the paper control were prepared with the same in-plane dimensions. The thicknesses of RHC-500-16 and the paper control were approximately 0.355 and 0.35 mm, respectively. Both samples were placed on a heating stage maintained at 100 °C and evaluated after 30 min under the same testing conditions. As shown in Figure 5b,c, the central surface temperatures were 75.2 °C for RHC-500-16 and 101.1 °C for the paper control. Because the density and intrinsic thermal properties of the paper control were not independently measured, this comparison is interpreted as a qualitative demonstration of thermal-barrier behavior rather than a quantitative comparison of intrinsic thermal conductivity.

To further examine the transient thermal response of RHC-500-16, the upper-surface temperature of the film was continuously monitored on heating stages maintained at 100 and 350 °C (Figure 5d–f). At 100 °C, the film temperature gradually increased and approached a fitted steady-state value of 77.23 °C, whereas at 350 °C the corresponding steady-state value was 267.41 °C. The coefficients of determination (R^2^) were 0.998 and 0.996, respectively, indicating good agreement between the fitted curves and the experimental data.

The first derivatives of the fitted curves were further used to evaluate the heating-rate evolution. At a stage temperature of 100 °C, the heating rate reached its maximum at approximately 16.5 s and decreased to nearly zero by approximately 160.3 s as the surface temperature approached a steady state. At 350 °C, the heating rate was highest during the initial stage and then progressively decreased with time. These results provide additional evidence of delayed heat transfer through the RHC-500-16 film under the present testing conditions.

Figure 5g shows the qualitative response of RHC-500-16 to the outer flame of an alcohol burner. Localized glowing occurred during the 10 s exposure, but sustained sample flaming was not observed. Visible glowing ceased within approximately 1 s after the burner was removed, and the film retained its gross shape in the final image. These observations describe only the qualitative response of the film under the present test conditions and should not be interpreted as evidence of standardized fire resistance or flame-retardant performance.

Rice-husk biochar/polymer composites have been investigated for their mechanical, thermal, and fire-related properties [32,33]. The RHC/PVDF film studied here likewise uses an abundant agricultural residue but is produced as a thin composite film by blade coating. This route avoids processing steps such as freeze-drying or vacuum-assisted forming and may therefore support scalable film production.

### 3.6. Proposed Thermal-Transport Interpretation and Study Boundaries

Heat transfer in porous carbon composites can proceed through the solid framework, the gas-filled pores, interfaces, and thermal radiation. The measured conductivity therefore reflects both pore morphology and the structure of the carbon phase.

As the pyrolysis temperature increased from 300 to 500 °C, progressive decomposition of lignocellulosic components produced a relatively uniform porous carbon framework. The mesopores and other accessible pores may restrict gas transport and limit gaseous heat conduction. Raman analysis also revealed changes in the structural disorder of the carbon phase. Such structural changes may influence solid-state heat transport.

When the pyrolysis temperature increased to 700 °C, the films instead showed coarser agglomeration, larger SEM-visible voids, and altered accessible mesopore characteristics. Although RHC-700-16 exhibited a higher Raman I_D_/I_G_ ratio than RHC-500-16, its thermal conductivity was also higher. This observation indicates that Raman-derived carbon disorder alone does not govern the thermal conductivity of these composite films.

Within the tested samples, thermal conductivity was therefore associated with the combined effects of pore characteristics, film morphology, interparticle contacts, and carbon structure rather than with any single structural parameter. RHC-500-16 provided the lowest measured thermal conductivity while retaining an intact film and a finer film morphology.

## 4. Conclusions

Blade coating produced intact RHC-500-16 and RHC-700-16 composite films, whereas RHC-300-16 did not form an intact self-supporting film. Consequently, the film-level morphology and thermal-performance comparison in this study was limited to the 500 and 700 °C samples. RHC-700-16 also fractured during the qualitative bending demonstration. The study examined how pyrolysis temperature affected char structure, film morphology, and thermal transport.

Increasing the pyrolysis temperature promoted lignocellulose decomposition and the formation of disordered aromatic carbon. At 500 °C, RHC-500-16 exhibited a BET specific surface area of 3.1347 m^2^·g^−1^, a total pore volume of 0.007716 cm^3^·g^−1^, and an average BJH pore diameter of 33.30 nm, compared with 1.9471 m^2^·g^−1^, 0.003153 cm^3^·g^−1^, and 21.66 nm for RHC-700-16, respectively. SEM observations additionally showed coarser agglomerates and larger visible voids in RHC-700-16, while Raman analysis indicated increased carbon disorder with increasing pyrolysis temperature.

RHC-500-16 had a room-temperature thermal conductivity of 0.059 W·m^−1^·K^−1^ and exhibited a lower surface temperature than the paper control in the qualitative heating-stage experiment under the tested conditions. Additional transient heating measurements showed that the surface temperature approached fitted steady-state values of 77.23 and 267.41 °C on heating stages maintained at 100 and 350 °C, respectively. The use of an abundant feedstock and a blade-coating process also supports further development of biomass-derived insulation films.

Within the tested conditions, these results link pyrolysis temperature to char structure, film morphology, and composite thermal conductivity. Future work should quantify tensile properties, bending resistance, cyclic mechanical durability, long-term thermal stability, moisture sensitivity, fire performance under standardized tests, and interfacial compatibility under relevant service conditions.

## Figures and Tables

**Table 1:** Mass yield of RHC after pyrolysis.

Sample	Initial Mass (g)	Mass After Carbonization (g)	Yield (%)
RHC-300	5.02	2.78	55.38
RHC-500	5.03	1.79	35.59
RHC-700	5.01	1.72	34.33

**Table 2:** Main FTIR absorption bands and their assignments for RH and RHC samples.

Wavenumber (cm^−1^)	Vibrational Assignment	Interpretation
3347.47	O–H stretching	Hydroxyl groups and adsorbed water; weakening/disappearance after carbonization reflects dehydration and loss of hydroxyl-containing groups
2916.55	Aliphatic C–H stretching	Aliphatic structures in lignocellulosic components; disappearance after carbonization indicates decomposition of aliphatic groups
1630.37	H–O–H bending vibration	Mainly associated with adsorbed water; weakening after carbonization reflects dehydration and reduced water adsorption
1072.23	Asymmetric Si–O–Si stretching	Siloxane bonding associated with silica-containing structures
799.73	Symmetric Si–O–Si stretching	Siloxane bonding associated with silica-containing structures
461.56	Si–O bending	Silica-related bonding vibration

**Table 3:** Elemental composition and atomic ratios of RH and RHC.

Sample	Elemental Analysis					Atomic Ratio		
	C (%)	H (%)	O (%)	N (%)	S (%)	H/C	O/C	(N + O)/C
RH	40.18	5.32	42.28	0.54	0.00	1.58	0.79	0.80
RHC-300	47.65	3.96	28.19	0.81	0.02	0.99	0.44	0.46
RHC-500	54.04	2.09	15.15	0.84	0.11	0.46	0.21	0.22
RHC-700	54.50	1.00	14.47	0.83	0.12	0.22	0.20	0.21

**Table 4:** Thermal conductivity and relevant thermal parameters.

Sample	Density (g cm^−3^)	Thermal Diffusivity (mm^2^·s^−1^)	Specific Heat (J·g^−1^·K^−1^)	Thermal Conductivity (W·m^−1^·K^−1^)
RHC-500-16	0.66	0.14	0.64	0.059
RHC-700-16	0.79	0.15	0.67	0.079

## Data Availability

The data presented in this study are available upon request from the corresponding author.

## Abbreviations

The following abbreviations are used in this manuscript:

RH	Rice husk
RHC	Rice husk char
PVDF	Polyvinylidene fluoride
NMP	N-Methyl-2-pyrrolidone
